# A Prospective Comparative Study of Using Ultrasonography, 4D-CT and Parathyroid Dual-Phase Scintigraphy with SPECT in Patients with Primary Hyperparathyroidism

**DOI:** 10.3390/diagnostics11112006

**Published:** 2021-10-28

**Authors:** Kalevi Kairemo, Aaron C. Jessop, A. Hans Vija, Xinhong Ding, Don Spence, S. Cheenu Kappadath, Homer A. Macapinlac

**Affiliations:** 1 Department of Nuclear Medicine, The University of Texas MD Anderson Cancer Center, Houston, TX 77030, USA; hmacapinlac@mdanderson.com; 2 Department of Theragnostics, Docrates Cancer Center, 00180 Helsinki, Finland; 3 Section of Nuclear Medicine, Department of Radiology and Radiological Sciences, Vanderbilt University Medical Center, Nashville, TN 37203, USA; aaron.jessop@vumc.org; 4 Siemens Medical Solutions USA, Inc. Molecular Imaging, Hoffman Estates, IL 60192, USA; hans.vija@siemens-healthineers.com (A.H.V.); xinhong.ding@siemens-healthineers.com (X.D.); don.spence@siemens-healthineers.com (D.S.); 5 Department of Medical Physics, The University of Texas MD Anderson Cancer Center, Houston, TX 77030, USA; skappadath@mdanderson.org

**Keywords:** parathyroid imaging, 4D-CT, SPECT/CT, ^99m^Tc -MIBI, hyperparathyroidism, parathyroid adenoma, ultrasound, xSPECT

## Abstract

Thirty-one consecutive patients were included in this study who were planned for parathyroidectomy due to primary hyperparathyroidism. They were studied with US, 4D-CT and dual-phase scintigraphy including SPECT/CT, and possible adenomas were identified in each imaging modality. Imaging data were quantified with US, CT and SPECT. Parathyroidectomies were performed as minimally invasive according to preoperative imaging findings. A total of 16 adenomas were found in 15 patients, and the surgery was negative in four patients. The imaging results were compared with each other and correlated to histology findings and blood biochemistry (S-Ca and P-PTH). Quantitative SPECT found a strong correlation between the quantification methods—Conjugate Gradient with Attenuation and Scatter Correction with a zone map (CGZAS) and Conjugate Gradient with Attenuation and Scatter Correction (CGAS)—measured as SUVmax and kBq/mL. However, a statistically significant correlation between the quantitative parameters (CGZAS and CGAS) and serum biomarkers (S-PTH and S-Ca) was not observed. The sensitivities of the imaging methods were calculated using histopathology as a gold standard. SPECT/CT demonstrated 93% sensitivity, 4D-CT 93% sensitivity and ultrasonography 73% sensitivity. The imaging methods were compared with each other using parathyroid regions because findings and locations varied between the modalities. Our prospective study supports that quantitative SPECT/CT is useful for presurgical assessment of primary hyperparathyroidism.

## 1. Introduction

Primary hyperparathyroidism is hyperfunction of parathyroid glands characterized by overproduction of parathyroid hormone. This elevates calcium concentrations in the blood circulation.

Minimally invasive surgical treatment is possible, if preoperative imaging has been performed successfully. Preoperative imaging consists of parathyroid scintigraphy, ultrasonography, 4D-CT or PET techniques. Parathyroid scintigraphy can be performed in multiple ways, such as a dual-tracer subtraction method or as dual-phase study. According to a recent meta-analysis, SPECT/CT is better than SPECT and planar scintigraphy; the sensitivities based on 18 articles were 84% (95% CI:78–90%) for SPECT/CT, for SPECT alone 66% (95% CI:57–74%), and for planar scintigraphy 63% (95% CI:51–74%) [1]. 

In a prospective Danish study of 108 patients, subtraction planar scintigraphy and planar dual phase scintigraphy were compared with 4D-CT and ultrasonography, and the sensitivities were 93%, 65%, 58% and 57%. The dual-tracer subtraction study was performed using the pinhole technique [2]. 

Standardized quantitative SPECT imaging with xSPECT Bone (TM) using CT-derived anatomical delineation for tissue boundary differentiation showed promise in assessment of symptomatic hip and knee joint prostheses, where quantification with 99mTc-dicarboxypropandiphosphate (DPD) allows for best discrimination between loose and stable prostheses among all of the evaluated methods [3,4]. In another use case in bone imaging for prostate and breast cancer, xSPECT Bone yielded an SUV-threshold for sensitivity allowing to distinguish benign and malignant lesions [3,4]. 

Here, we present our findings in patients who were studied using ultrasonography, 4D-CT and parathyroid dual-phase scintigraphy with SPECT/CT prior to potential minimally invasive parathyroidectomy. 

## 2. Materials and Methods 

### 2.1. Patients Characteristics

Prospectively, 31 consecutive patients were included with primary hyperparathyroidism prior to possible parathyroidectomy. The patient population consisted of 12 male (with 13 adenoma suspicions) and 19 female (with 21 adenoma suspicions) patients aging from 31 to 85 years. They were studied with US, 4D-CT and dual-phase scintigraphy including SPECT/CT with ^99m^Tc-MIBI. Patient characteristics are listed in Table 1, these patients underwent routine parathyroid ^99m^Tc-MIBI imaging for elevated PTH level and ultrasonography for existing abnormal parathyroid glands. In the Table 1 patient demographics (gender, age) and laboratory values (calcium, parathyroid hormone), imaging, and operative and pathological results and outcomes are listed. Parathyroidectomies were performed as minimally invasive guided by preoperative imaging findings. 

Imaging data were quantified using dimensions (volumes) in US and CT. For patients undergoing parathyroidectomy, extirpated tissues were weighed and analyzed for histopathology. 

The imaging results were compared with each other, correlated to histology findings and blood biochemistry (S-Ca, normal range 7.6–10.4 µg/L (1.9–2.6 mmol/L) and P-PTH, normal range 10–65 ng/L). The imaging findings were evaluated using histopathology as a gold standard. The imaging characteristics were analyzed on a patient-by-patient basis and based on the number of surgically excised tissue specimens. 

This study was performed in accordance with the guidelines of the MD Anderson Institutional Review Board (IRB grant protocol# 2012-00036240).

### 2.2. Imaging Examinations

All patients received an intravenous injection of 740 MBq of ^99m^Tc-MIBI. Early parathyroid imaging was obtained 15–20 min post injection, and a delayed parathyroid imaging was obtained 75–90 min post the injection. SPECT/CT integrated imaging was performed around 30 min immediately after the early planar image and before the delayed planar image. The imaging acquisition was using Siemens Symbia T16 SPECT/CT gamma camera with VA63 software, allowing for advanced and list mode data recording in research mode. The Symbia T16 imaging system and local dose calibrators were calibrated with a 111 MBq nominal 3% NIST traceable ^57^Co calibrated sensitivity source (CSS) ensuring standardization and cross calibration of sensitivities. A 256 × 256 matrix with 2.39 × 2.39 mm^2^ pixel was used and 128 projections were acquired over 360° with body-contour orbit for 20 s per projection with a 15% photopeak window centered at 140 keV and a 15% adjacent lower scatter window. Imaging data were reconstructed using a three-dimensional iterative algorithm, a functional identical prototype of xSPECT Quant and xSPECT Bone. However, the data were acquired with VA63A and required reconstruction with research software. Since this study, the prototype has become commercially available [5,6]. CGAS is a base reconstruction algorithm based on the Conjugate Gradient method (unlike Flash 3D, which is an OSEM method), using the Mighell modified chi-squared merit function for Poisson statistics [7]. “A” refers to Attenuation Correction and “S” to Scatter Correction. CGZAS includes delineation of tissue boundaries (“zones”) where the “Z” refers to zoning [5,6]. In this case, the zoning is for CT-derived segmentation of the parathyroid, enabling the reconstruction to be parathyroid specific, yet data driven. The 3D segmentation in this model is human based and not automated. Both methods are quantitative with standardized sensitivity calibration via the CSS. CGZAS has an inherent, but no dedicated mitigation for the partial volume, and both methods assume absence of tomographic inconsistency due to, e.g., motion. Resolution recovery coefficients can be estimated from the white paper [6]. The reconstruction parameters are set to 48 updates and 1 sub-set, as recommended. The zoning reconstruction should provide a more stable estimate of activity uptake across the population as compared to basic xSPECT Quant, but it may not be sufficient to control of variation due to noise, motion, and partial volume. 

Images were smoothed with a three-dimensional spatial Gaussian filter with a Full-Width-at-Half-Maximum (FWHM) of 10 mm, corresponding to a nominal matched filter given the resolution of the LEHR collimator and typical orbit radii. 

CT acquisition parameters were as follows: tube peak voltage of 130 kVp and tube current modulation with CareDose4D was used with a reference mAs of 90 (the average mAs was 25.8, ranged between 24–27 mAs). For CT data reconstruction, a 3 mm slice thickness with 2 mm slice increment was used with the B31 filter. Both SPECT and CT 3 mm slices were generated and were transferred to a picture archiving and communication system after generation of DICOM files. SPECT/CT images were fused using the Syngo software. 

### 2.3. Image Analysis

The imaging results were evaluated by visual analysis and the uptake value of lesions were judged by semi-quantitative visual analysis. SPECT/CT images were analyzed by two experienced nuclear medicine physicians (KK; ACJ) who were blinded to the laboratory, surgical, and pathological results. Abnormal ^99m^Tc-MIBI uptake was considered to be positive on visual analysis, and the different uptake value of each lesion was graded to three levels through the calculation of the tumor to background ratio (TBR) for the delayed phase. These areas were scored for activity on a three-point scale: 1 = weak uptake (1 < TBR ≤ 2), 2 = moderate uptake (2 < TBR ≤ 3), 3 = high uptake (TBR > 3). A positive parathyroid imaging result was increased uptake of ^99m^Tc-MIBI on the delayed image compared to the early-stage image, with precise localization of the focus on the delayed planar image by SPECT/CT; negative parathyroid scan had no ^99m^Tc-MIBI increased uptake on the delayed image compared to background.

US and 4D-CT investigations were part of institutional routine protocols in these patients and the studies were analyzed by experienced radiologists in charge. The imaging findings were compared within different modalities: volumes were measured with US, lesion diameter(s) by 4D-CT and activity concentrations by SPECT.

## 3. Results

**Figure 1:**
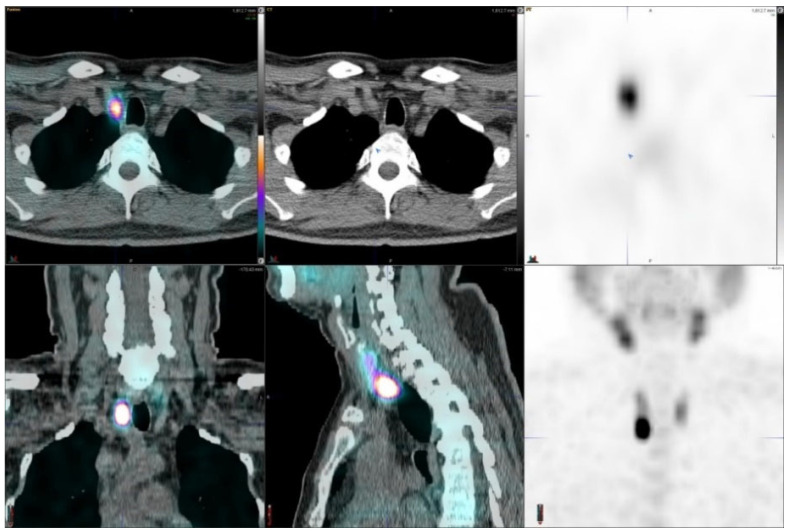
A 55-year-old male with hyperparathyroidism, right inferior parathyroid adenoma, it weighed 2.4 g. His S-PTH concentration was 162 ng/L and S-Ca 10.9 µg/L. The SUVmax values were 21.8 (CGZAS) and 16.7 (CGAS), respectively. Visually, this was a high uptake (TBR > 8) and the lesion diameter on CT was 18 mm.

Three out of thirty-one hyperparathyroidism patients had multiple lesions on the imaging studies, eight patients had no adenomas. A total of 24 adenomas were found in 21 patients (Table 1). Figure 1 illustrates a patient with a large right inferior parathyroid adenoma which demonstrated a high uptake. This adenoma was removed surgically and it weighed 2.4 g. 

**Figure 2:**
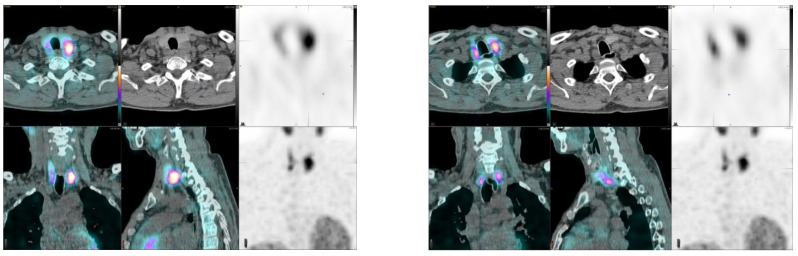
A 55-year-old male with hyperparathyroidism, right inferior adenoma, left parathyroid carcinoma. His S-PTH concentration was 478 ng/L and S-Ca 11.1 µg/L. The SUVmax values of the right-sided adenoma were 6.3 (CGZAS) and 3.8 (CGAS), respectively. Visually, this uptake was weak (1 < TBR < 2) and lesion diameters on CT were 21 mm × 24 mm. The carcinoma on the left demonstrated higher SUVmax values than the adenoma: 8.3 (CGZAS) and 7.3 (CGAS), respectively. Visually this uptake was moderate (2 < TBR < 3) and the lesion diameters on CT were 25 mm × 30 mm. This patient was not operated because of MEN syndrome.

Figure 2 demonstrates two possible adenomas, a weak uptake on the right and a higher uptake on the left. In the surgery, the left side lesion turned out be parathyroid carcinoma. 

**Figure 3:**
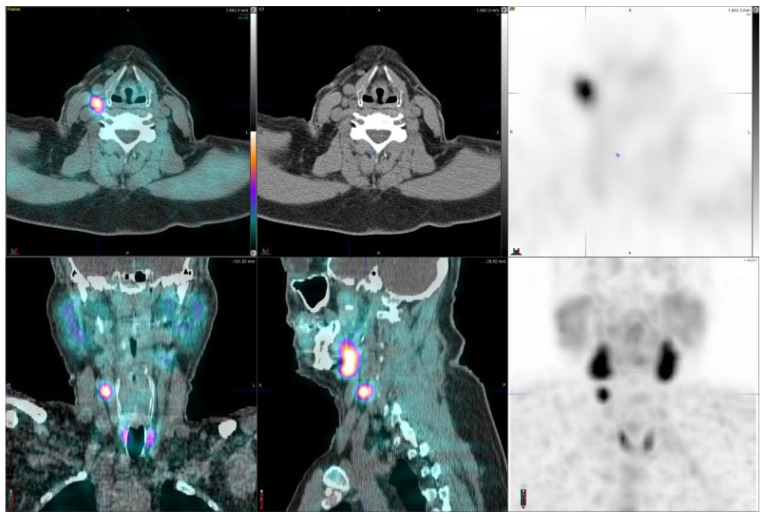
Parathyroid adenoma, in the right lateral mid neck, weighing 1.54 g. This 53-year-old male had S-PTH concentration of 125 ng/L and S-Ca of 11.0 µg/L. The SUVmax values of the right-sided adenoma were 7.8 (CGZAS) and 4.7 (CGAS), respectively. Visually, this uptake was high (TBR > 3) and lesion diameters on CT were 9 mm × 21 mm.

**Figure 4:**
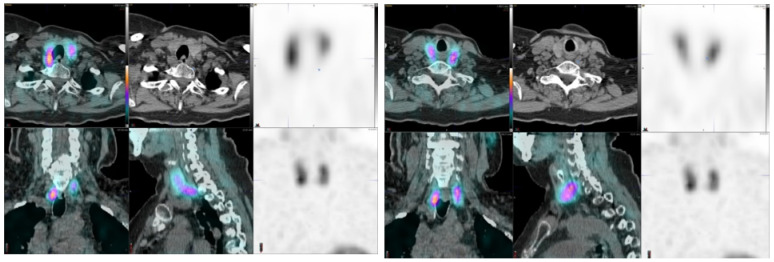
Parathyroid adenoma, on the right (inferior), on the left (superior). This 69-year-old female patient was not operated. Her S-PTH concentration was 113 ng/L and S-Ca 11.0 µg/L. The SUVmax values of the right-sided adenoma were 10.4 (CGZAS) and 7.8 (CGAS), respectively. Visually, this uptake was moderate (2 < TBR < 3) and lesion diameters on CT were 11 mm × 24 mm. The adenoma on the left demonstrated slightly higher SUVmax values: 12 (CGZAS) and 9.3 (CGAS), respectively. Visually this uptake was weaker, but moderate (2 < TBR < 3) and the lesion diameters on CT were 17 mm × 38 mm.

**Figure 5:**
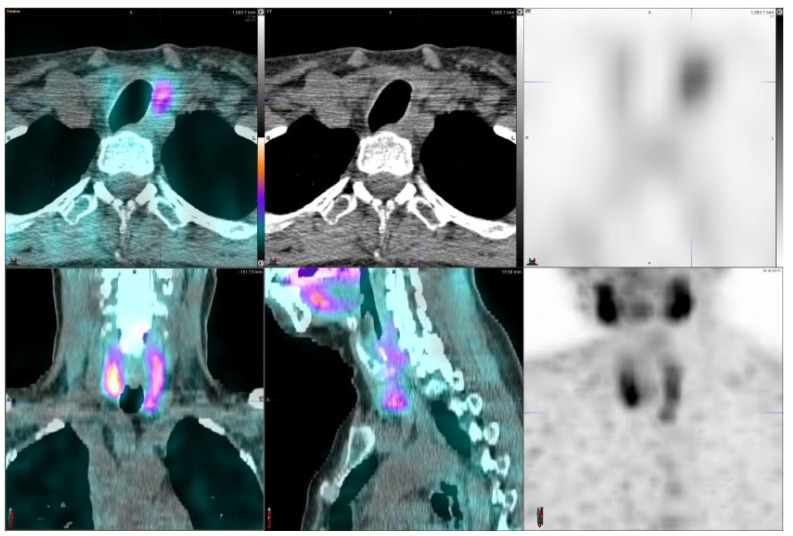
A 65-year old female with hyperparathyroidism. A left inferior parathyroid adenoma, weighing 123 mg and an intrathyroid nodule on the right are seen. Her S-PTH concentration was 154 ng/L and S-Ca 10.9 µg/L. The SUVmax values of the left-sided adenoma were 5.1 (CGZAS) and 3.1 (CGAS), respectively. Visually, this uptake was weak (1 < TBR < 2) and lesion diameter on CT was 7 mm. The intrathyroid nodule demonstrated higher uptake. This is a potential pitfall in adenoma imaging, but can be avoided by multimodality practice.

Figure 3 shows an adenoma with a high uptake in the right lateral mid neck region; this tumor weighed 1.5 g. Figure 4 demonstrates two possible adenomas, a moderate uptake on the right and a weaker uptake on the left. Figure 5 illustrates a left inferior parathyroid adenoma with a weak uptake and an unusual right intrathyroid nodule. The extirpated left parathyroid adenoma weighed 123 mg.

One patient demonstrated a parathyroid malignancy (Figure 2). 

A total of 19 patients were operated on while 12 patients had no surgery. A total of 16 lesions were found surgically in 15 patients. Four patients who were operated on did not have parathyroid adenomas localized surgically (Table 1). SPECT/CT turned out to be the most accurate, missing only one adenoma found surgically. US findings differed from those of SPECT/CT in seven lesions; US missed six adenomas as compared to SPECT and found one adenoma which SPECT missed. CT findings differed from those of SPECT/CT in five lesions; four new adenomas were found, and CT missed one adenoma as compared to SPECT/CT (Table 1). The sensitivities on a patient-by-patient basis as compared to surgery were for SPECT/CT 93% (14/15), for 4D-CT 93% (14/15) and for ultrasonography 73% (11/15), and per lesions SPECT/CT 94% (15/16), for 4D-CT 94% (15/16) and for ultrasonography 75% (12/16), respectively. Specificities cannot be calculated because there were only four patients who were not found to have adenomas surgically. Actually, in three of these methods, SPECT/CT, 4D-CT and US, demonstrated small adenoma, which were not found in the surgery; thus, only one patient had a true negative finding. The 4D-CT method suspected five adenomas in normal parathyroid glands, whereas SPECT/CT and US demonstrated three false positive forementioned findings. SPECT/CT missed one adenoma which was detected both by US and CT (Table 1).

**Figure 6:**
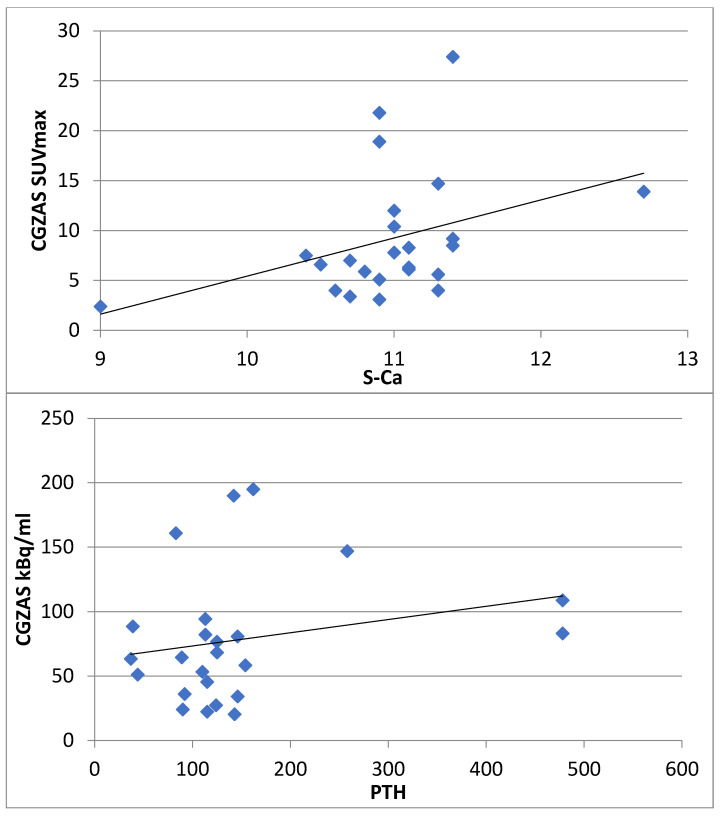
The correlations between serum parathormone (**upper**) and calcium (**lower**) concentration and quantitative SPECT. The correlation is weak, *p* < 0.05.

We also analyzed the quantitative SPECT data and found only a weak correlation between the quantification methods CGAS and CGZAS measured as SUVmax and kBq/mL between serum biomarkers (S-PTH and S-Ca) (Figure 6). The Pearson correlation coefficients were 0.091 between CGZAS SUVmax and S-PTH, and 0.416 between CGZAS SUVmax and S-Ca (*n* = 25). Correlation coefficients were 0.269 between CGZAS kBq/mL and S-PTH, and 0.440 between CGZAS kBq/mL and S-Ca (*n* = 25). For example, the Wilcoxon test for paired samples gave a statistical significance of *p* = 0.037 between CGZAS SUVmax and S-Ca, indicating that the correlation is weak.

## 4. Discussion

Parathyroid is the last macroanatomy finding, discovered as late as the 1880s in Sweden [8]. Primary hyperparathyroidism is a common endocrine disorder globally, affecting approximately 1 in 500 women and 1 in 2000 men, and the primary treatment is surgery [9]. Exact preoperative localization is essential to perform focused or minimally invasive surgery for primary hyperparathyroidism. Normal parathyroid glands are too small to be visualized by external imaging (Figure 1, Figure 2, Figure 3, Figure 4 and Figure 5), but parathyroid enlargement often makes them visible. In the patients we studied here, the smallest visualized adenomas weighed less than 0.12 grams. 

Preoperative diagnosis for adenomas is best performed by SPECT methods: the two most common imaging agents are technetium-99m-MIBI [10,11,12] and Tc-99m tetrofosmin [13]. Optimal parathyroid scintigraphy requires an understanding of the anatomy and physiology of the parathyroid glands as well as the characteristics of the SPECT tracers. Enlarged parathyroid glands may be found near the thyroid gland or outside their expected locations [14]. Typical abnormal scintigraphic patterns may be described as focal or multifocal, usual or ectopic in location, and associated with a normal or abnormal thyroid gland. For example, Figure 2 demonstrates multifocal finding, Figure 3 an ectopic adenoma and Figure 4 a rare intrathyroid nodule and a solitary adenoma. An incidental finding of a parathyroid carcinoma as in this study is extremely rare, but those can be associated with MEN disorders.

According to larger series, SPECT/CT is usually better than planar techniques [10]. According to a recent meta-analysis consisting of twenty-three articles including 1236 patients with primary hyperparathyroidism the pooled detection rate of ^99m^Tc-MIBI SPECT/CT in the preoperative planning of patients with primary hyperparathyroidism was per patient-based 88% (95% CI; 82–92%) and per lesion-based analysis 88% (95% CI; 82–94%) [15].

Planar techniques, however, can be improved up to higher than 90% sensitivity, by introducing multiphase imaging, dual-tracer techniques or by pinhole imaging [2], but this method was not compared with SPECT/CT. In another study, the utility of delayed neck and thorax SPECT/CT over dual-phase ^99m^Tc-MIBI planar scintigraphy is that it can identify and locate a parathyroid tumor in about more than 70% of patients in primary hyperparathyroidism and perform required surgical planning [16].

Planar imaging is no longer recommended as a single modality while SPECT/CT and 4D-CT exist. In prospective studies, sensitivities of 93% and specificities of 99% for SPECT/CT were reported [1]. ^99m^Tc-MIBI–SPECT/CT image fusion is known to be superior to CT or MIBI-SPECT alone for more than 10 years [17]. In a study of 58 patients, combining the data of SPECT/CT and 4D-CT increased sensitivity to 88%, specificity to 100%, and accuracy to 89% [18]. In a recent study, the sensitivity in preoperative localization for parathyroid adenomas was further improved by adding contrast-enhanced CT to ^99m^Tc-MIBI-SPECT/CT; for example, in 140 operated patients with uniglandular disease, the sensitivity increased from 86.4% to 93.6% (*p* = 0.021) and the specificity from 96.2% to 97.9% [19]. 

In our study, we were able to compare the diagnostic performance of ultrasonography, 4D-CT and SPECT/CT combined with CT. In our material, dual-phase planar scintigraphy did not reveal any additional lesions as compared to SPECT/CT, therefore only the imaging modalities (US, CT, SPECT) were compared with each other. SPECT/CT demonstrated 93% sensitivity, 4D-CT 93% sensitivity, and ultrasonography 73% sensitivity. These numbers are in concordance with the results of the two meta-analyses [1,15] and with the later studies [18,19,20]. On the other hand, our sample size is too small for further subgroup analyses. CT had five false positive findings, whereas SPECT/CT demonstrated three questionable false positive findings seen on every other imaging modality. There was a total of 12 patients who were not operated on, mainly due to SPECT findings or lack of symptoms. Therefore, it is unclear if there were any false negative findings.

In this prospective study, another main goal was to use absolute concentration calculations, based on standardized uptake value (SUV) concentrations. New quantification methods were available for SPECT (CGZAS and CGAS (SUVmax, kBq/mL)), but these did not characterize the hyperparathyroidism (Figure 6). This lack of strong positive correlation between blood chemistry (S-Ca and S-PTH) and quantitative SPECT concentrations is probably more due to low specificity and due to parathyroid physiology than SPECT imaging data. However, for this condition imaging is mandatory, and the disease can seldom be fully characterized by in vitro laboratory criteria. In a recent large study consisting of more than 2000 patients, both preoperative calcium and parathormone turned out to be poor predictors of adenoma size and multiglandular disease [21]. PTH related hypercalcemia is most commonly caused by primary hyperparathyroidism, when adenomas are most often eutopic, i.e., have parathyroid location when imaged with ^99m^Tc-MIBI- SPECT/CT, but several adenomas with ectopic locations have been published in the literature [22]. Parathyroid hormone related hypercalcemia may also be associated with multiple endocrine neoplasia (type 1) [22]. We show an ectopic adenoma in Figure 3, and a syndromic (MEN) hypercalcemia patient in Figure 2. In a recent study consisting of 107 patients, SPECT was superior to US to detect ectopic disease, even though they found US superior to SPECT in detecting adenomas preoperatively [20]. Anecdotally, studies have shown that larger parathyroid adenomas are associated with lower vitamin D levels, which are more likely to be detected by ^99m^Tc-MIBI-SPECT/CT [23].

In our material we had special cases, such as intrathyroid adenomas, which easily can be considered as parathyroid adenomas. CT is essential in the differential diagnosis. We also had a patient with both malignant and benign parathyroid tumor. There was no essential difference in the tracers’ behavior. The observed (CGZAS method) concentration in the carcinoma was SUVmax 8.3 and 109 kBq/mL and in the adenoma SUVmax 6.3 and 83.3 kBq/mL. The highest observed (CGZAS method) concentration in a surgically removed adenoma (2400 mg) was SUVmax 21.8 and 195 kBq/mL and lowest concentration SUVmax 1.2 and 13.3 kBq/mL in in an adenoma of 585 mg, i.e., higher than 15-fold difference. From our study, it is obvious that SUV values do not play an essential role in the differential diagnosis. ^18^F-fluorocholine-positron PET/CT has recently been introduced for detecting hyperfunctioning parathyroid glands in hyperparathyroidism [24]. SUVmax was known to be higher in parathyroid adenomas than in hyperplasia in 76 hyperparathyroidism patients with verified histopathology imaged with ^18^F-fluorocholine PET/CT, but this finding was not statistically significant [25]. In practice, PET methods are accurate, because of their sensitivity, multiple tracers are available, such as [^18^F]fluorocholine) PET/CT and L-[methyl-^11^C]methionine ([^11^C]MET) PET/CT [26] or O-(2-[^18^F]fluoroethyl)-L-tyrosine ([^18^F]FET) [27]. A recent meta-analysis favors [^18^F]fluorocholine) PET/CT in the diagnosis of primary hyperparathyroidism [28].

The original idea of this prospective study was to test SPECT quantitatively in this condition where nuclear medicine plays a major role in the diagnostic algorithm. The result of this was not known in advance, and from this pilot study we learned that the situation is complex. CGZAS may be a choice in primary hyperparathyroidism.

Overall, precise quantification may play a role in studying hyperparathyroidism, but for this larger populations should be investigated with various method-oriented cohorts. 

## 5. Conclusions

We conclude that dual-phase scintigraphy with SPECT/CT in patients with suspicion of primary hyperparathyroidism should be included in the preoperative imaging, as previously known from the meta-analyses and major studies with histology correlations. 

A larger cohort is needed to make further conclusions, but our findings support that SPECT/CT, preferably quantitatively, is an essential part of presurgical evaluation of primary hyperparathyroidism.

## Figures and Tables

**Table 1:** Patient characteristics. Age, gender, adenoma (lesion) site (left, right), total thyroid volume (mL), lesion volume in US (mL), lesion size in CT (mm), lesion weight in surgery (mg), lesion size in surgery (mm), S-PTH and S-Ca at the time of imaging diagnosis, CGZAS and CGAS as measured from xSPECT in (kBq/mL) and (SUVmax). Abbreviations: SPECT = single photon emission computed tomography, US = ultrasonography, CT = computed tomography, CGZAS and CGAS (see text), L = left, R = right, nd = not done, nm = not measured. Yellow color refers to an adenoma on SPECT, green color refers to a match in US, CT or surgery and red color to a mismatch in US or CT. Brown colors indicate dual lesions in same patient.

Age/Gender	Dgn/SPECT	Thyroid/US Volume	Lesion/ US Volume	Lesion/ CT Size	Lesion wt /Surgery	Lesion Size/Surgery	S-PTH	S-Ca	CGZAS kBq/mL	CGZAS SUVmax	CGAS kBq/mL	CGAS SUVmax
66/f	adenoma L	6.8	0.084	7 mm L	123 mg	10 mm	154	10.9	58.5	5.1	35.3	3.1
53/m	adenoma L	6.5	1.3	12 × 14 L	1854 mg	30 mm	83	10.9	161	18.9	83.1	9.8
36/f	adenoma L	11.2	0.43	0	nd	nd	39	10.5	88.7	6.6	65.8	4.9
65/f	adenoma R	9.1	0.36	13 × 14 R	585 mg	nm	118	10.2	13.3	1.2	25.7	2.3
55/m	adenoma R	12.7	1.6	18 mm R	2400 mg	30 mm	162	10.9	195	21.8	149	16.7
81/f	no adenoma	3.7	0.05	5 mm R	244 mg	27 mm	129	9.1				
62/f	adenoma R	8.8	2.4	12 mm R	2400 mg	34 mm	92	11.3	36.2	5.6	26.9	4.1
53/f	adenoma L	10.0	0	4 mm L	nd	nd	44	10.9	51.3	3.1	41.3	2.5
63/m	no adenoma	15.1	0	7 mm R	nd	nd	59	10.7				
59/f	no adenoma	nm	0	0	nd	nd	93	10.4				
60/f	double adenoma L		0.784	11 mm L	117 mg	nm	115	10.7	22.5	3.4	20.8	3.2
60/f	double adenoma L		0.216	11 mm L	155 mg	nm	115	10.7	45.6	7	36.2	5.5
72/f	adenoma L	10.1	0	10 × 17 L	nm	15 × 10	89	10.4	64.6	7.5	53.5	6.2
68/f	adenoma R	4.8	0.1	8 mm R	nd	nd	90	10.6	24.2	4	15.7	2.6
64/f	adenoma R	4.0	0.9	7 mm R	nd	nd	258	12.7	147	13.9	102	9.7
31/m	adenoma R	11.0	0.084	9 × 12 R	nd	nd	110	10.8	53.4	5.9	32.2	3.6
68/f	no adenoma	nm	0	0	nd	nd	44	10.1				
61/m	adenoma R	nm	nm	8 mm R			37	11.4	63.5	8.5	33.4	4.5
73/m	no adenoma	12.0	0	0	nd	nd	72	11.2				
58/f	no adenoma	17.4	0	5 × 8 R	nd	nd	95	10.6				
56/f	adenoma R	nm	nm	6 mm R	nd	nd	143	9.0	20.5	2.4	14.3	1.7
70/m	adenoma L	11.6	0.158	32.18.26 R	3680 mg	23 × 21	142	11.4	190	27.4	143	20.7
68/m	adenoma R	19.2	0	13 × 13 R	736 mg	10 × 7	124	11.1	27.5	6.1	29.5	4.9
73/f	adenoma L	7.5	0	8 × 18 L	478 mg	22 × 9	146	11.3	80.9	14.7	62	3.6
55/m	adenoma R	nm	nd	21 × 24 R	nd	nd	478	11.1	83.3	6.3	49.6	3.8
55/m	carcinoma L	nm	nd	25 × 30 L	nd	nd	478	11.1	109	8.3	96.2	7.3
69/f	adenoma L	18.8	1.52	17 × 38 R	nd	nd	113	11.0	94.5	12	73.9	9.3
69/f	adenoma R	18.8	0.972	11 × 24 L	nd	nd	113	11.0	82.3	10.4	61.4	7.8
85/f	no adenoma	6.2	0.113	6 × 12 L	nd	nd	97	10.6				
71/f	no adenoma	nm	0	nm R	nd	nd	109	10.6				
77/f	adenomaL	5.5	0	8 mmL	173 mg	10 × 6	125	11.4	76.8	9.2	43.5	5.2
68/m	no adenoma	22.8	1.9 diam	12 × 14 L	1130 mg	20 × 12	49	9.9				
53/m	adenoma L	nm	0	9 × 21 R	1540 mg	20 × 13	125	11.0	68.4	7.8	40.8	4.7
37/m	adenoma L	8.9	0	0	200 mg	12 mm	5	11.3	34.3	4	25.1	2.9

## Data Availability

The datasets generated during and/or analyzed during the current study are available from the corresponding author on reasonable request.

## References

[1] Wei W.J., Shen C.T., Song Z.L., Luo Q.Y. (2015). Comparison of SPECT/CT, SPET and planar imaging using ^99m^Tc-MIBI as independent techniques to support minimally invasive parathyroidectomy in primary hyperparathyroidism: A meta-analysis. Hell. J. Nucl. Med..

[2] Krakauer M., Wieslander B., Myschetzky P.S., Lundstrøm A., Bacher T., Sørensen C.H., Trolle W., Nygaard B., Bennedbæk F.N. (2016). A prospective comparative study of parathyroid dual-phase scintigraphy, dual-isotope subtraction scintigraphy, 4D-CT and ultrasonography in primary hyperparathyroidism. Clin. Nucl. Med..

[3] Braun M., Cachovan M., Kaul F., Caobelli F., Bäumer M., Vija A.H., Pagenstert G., Wild D., Kretzschmar M. (2021). Accuracy comparison of various quantitative [99mTc]Tc-DPD SPECT/CT reconstruction techniques in patients with symptomatic hip and knee joint prostheses. EJNMMI Res..

[4] Vija A.H., Bartenstein P.A., Froelich J.W., Kuwert T., Macapinlac H., Daignault C.P., Gowda N., Hadjiev O., Hephzibah J., Huang P. (2019). ROC study and SUV threshold using quantitative multi-modal SPECT for bone imaging. Eur. J. Hybrid Imaging.

[5] Vija A.H., Vija A.H. (2013). Introduction to the xSPECT Technology: Evolving Multi Modal SPECT to Become Context Based and Quantitative.

[6] Vija A.H. (2017). Characteristics of the xSPECT Reconstruction Method.

[7] Mighell K.J. (1999). Parameter Estimation in Astronomy with Poisson-distributed Data. I. The χγ2 Statistic. Astrophys. J..

[8] Sandström I. (1879). Om en ny körtel hos menniskan och åtskilliga däggdjur. Upsala Läkareförenings Förhandlingar.

[9] Shah S., Win Z., Al-Nahhas A. (2008). Multimodality imaging of the parathyroid glands in primary hyperparathyroidism. Minerva Endocrinol..

[10] Lavely W.C., Goetze S., Friedman K.P., Leal J.P., Zhang Z., Garret-Mayer E., Dackiw A.P., Tufano R.P., Zeiger M.A., Ziessman H.A. (2007). Comparison of SPECT/CT, SPET, and planar imaging with single- and dual-phase ^99m^Tc-MIBI parathyroid scintigraphy. J. Nucl. Med..

[11] Neumann D.R., Obuchowski N.A., Difilippo F.P. (2008). Preoperative ^123^I/^99m^Tc-MIBI subtraction SPET and SPET/CT in PHPT. J. Nucl. Med..

[12] Vu T., Schellingerhout D., Guha-Thakurta N., Sun J., Wei W., Kappadth S., Perrier N., Kim E., Rohren E., Chuang H. (2019). Solitary Parathyroid Adenoma Localization in Technetium Tc99m Sestamibi SPECT and Multiphase Multidetector 4D CT. Am. J. Neuroradiol..

[13] Hiromatsu Y., Ishibashi M., Nishida H., Okuda S., Miyake I. (2000). Technetium-99m Tetrofosmin Parathyroid Imaging in Patients with Primary Hyperparathyroidism. Intern. Med..

[14] Smith J.R., Oates M.E. (2004). Radionuclide imaging of the parathyroid glands: Patterns, pearls, and pitfalls. Radiographics.

[15] Treglia G., Sadeghi R., Schalin-Jäntti C., Caldarella C., Ceriani L., Giovanella L., Eisele D.W. (2016). Detection rate of 99m Tc-MIBI single photon emission computed tomography (SPECT)/CT in preoperative planning for patients with primary hyperparathyroidism: A meta-analysis. Head Neck.

[16] Qiu Z.-L., Wu B., Shen C., Zhu R.-S., Luo Q.-Y. (2014). Dual-phase 99mTc-MIBI scintigraphy with delayed neck and thorax SPECT/CT and bone scintigraphy in patients with primary hyperparathyroidism: Correlation with clinical or pathological variables. Ann. Nucl. Med..

[17] Wimmer G., Profanter C., Kovacs P., Sieb M., Gabriel M., Putzer D., Bale R., Margreiter R., Prommegger R. (2009). CT-MIBI-SPECT image fusion predicts multiglandular disease in hyperparathyroidism. Langenbeck’s Arch. Surg..

[18] Kedarisetty S., Fundakowski C., Ramakrishnan K., Dadparvar S. (2019). Clinical Value of Tc99m-MIBI SPECT/CT Versus 4D-CT or US in Management of Patients With Hyperparathyroidism. Ear Nose Throat J..

[19] Sandqvist P., Nilsson I.-L., Grybäck P., Sanchez-Crespo A., Sundin A. (2019). Multiphase Iodine Contrast-Enhanced SPECT/CT Outperforms Nonenhanced SPECT/CT for Preoperative Localization of Small Parathyroid Adenomas. Clin. Nucl. Med..

[20] Lu R., Zhao W., Yin L., Guo R., Wei B., Jin M., Zhou X., Zhang C., Lv X. (2021). Efficacy of ultrasonography and Tc-99m MIBI SPECT/CT in preoperative localization of parathyroid adenomas causing primary hyperthyroidism. BMC Med. Imaging.

[21] Naples R., Thomas J.D., Monteiro R., Zolin S.J., Timmerman C.K., Crawford K., Jin J., Shin J.J., Krishnamurthy V.D., Berber E. (2021). Preoperative Calcium and Parathyroid Hormone Values Are Poor Predictors of Gland Volume and Multigland Disease in Primary Hyperparathyroidism: A Review of 2000 Consecutive Patients. Endocr. Pract..

[22] Han C.H., Fry C.H., Sharma P., Han T.S. (2020). A clinical perspective of parathyroid hormone related hypercalcaemia. Rev. Endocr. Metab. Disord..

[23] Kandil E., Tufaro A.P., Carson K.A., Lin F., Somervell H., Farrag T., Dackiw A., Zeiger M., Tufano R.P. (2008). Correlation of Plasma 25-Hydroxyvitamin D Levels With Severity of Primary Hyperparathyroidism and Likelihood of Parathyroid Adenoma Localization on Sestamibi Scan. Arch. Otolaryngol.-Head Neck Surg..

[24] Michaud L., Burgess A., Huchet V., Lefèvre M., Tassart M., Ohnona J., Kerrou K., Balogova S., Talbot J.-N., Perie S. (2014). Is 18F-Fluorocholine-Positron Emission Tomography/Computerized Tomography a New Imaging Tool for Detecting Hyperfunctioning Parathyroid Glands in Primary or Secondary Hyperparathyroidism?. J. Clin. Endocrinol. Metab..

[25] Beheshti M., Hehenwarter L., Paymani Z., Rendl G., Imamovic L., Rettenbacher R., Tsybrovskyy O., Langsteger W., Pirich C. (2018). 18F-Fluorocholine PET/CT in the assessment of primary hyperparathyroidism compared with 99mTc-MIBI or 99mTc-tetrofosmin SPECT/CT: A prospective dual-centre study in 100 patients. Eur. J. Nucl. Med. Mol. Imaging.

[26] Ovčariček P.P., Giovanella L., Gasset I.C., Hindié E., Huellner M.W., Luster M., Piccardo A., Weber T., Talbot J.-N., Verburg F.A. (2021). The EANM practice guidelines for parathyroid imaging. Eur. J. Nucl. Med. Mol. Imaging.

[27] Krakauer M., Kjaer A., Bennedbæk F.N. (2016). 18F-FET-PET in Primary Hyperparathyroidism: A Pilot Study. Diagnostics.

[28] Bioletto F., Barale M., Parasiliti-Caprino M., Prencipe N., Berton A.M., Procopio M., Deandreis D., Ghigo E. (2021). Comparison of the diagnostic accuracy of 18F-Fluorocholine PET and 11C-Methionine PET for parathyroid localization in primary hyperparathyroidism: A systematic review and meta-analysis. Eur. J. Endocrinol..

